# Optimizing Surgical Antibiotic Prophylaxis in the Era of Antimicrobial Resistance: A Position Paper from the Italian Multidisciplinary Society for the Prevention of Healthcare-Associated Infections (SIMPIOS)

**DOI:** 10.3390/pathogens14101031

**Published:** 2025-10-11

**Authors:** Massimo Sartelli, Francesco M. Labricciosa, Beatrice Casini, Francesco Cortese, Monica Cricca, Alessio Facciolà, Domitilla Foghetti, Matteo Moro, Angelo Pan, Daniela Pasero, Giuseppe Pipitone, Giancarlo Ripabelli

**Affiliations:** 1 Department of General Surgery, Macerata Hospital, 62100 Macerata, Italy; 2 Global Alliance for Infections in Surgery, 62100 Macerata, Italy; labricciosafrancesco@gmail.com; 3 Department of Translational Research and New Technologies in Medicine and Surgery, University of Pisa, 56126 Pisa, Italy; beatrice.casini@med.unipi.it; 4 Emergency Surgery Unit, San Filippo Neri Hospital, 00135 Roma, Italy; francescocortese@gmail.com; 5 Unit of Microbiology, The Greater Romagna Area Hub Laboratory, 47522 Cesena, Italy; monica.cricca3@unibo.it; 6 Department of Medical and Surgical Sciences (DIMEC), University of Bologna, 40138 Bologna, Italy; 7 Department of Biomedical and Dental Sciences and Morphofunctional Imaging, University of Messina, 98125 Messina, Italy; alessio.facciola@unime.it; 8 Department of Surgery, “Santa Croce” Hospital, 61032 Fano, Italy; domitilla.foghetti@gmail.com; 9 Infection Prevention and Control Team, IRCCS Ospedale San Raffaele, 20132 Milan, Italy; moro.matteo@hsr.it; 10 Unit of Infectious Diseases, ASST Cremona, 26100 Cremona, Italy; angelo.pan@asst-cremona.it; 11 Department of Medicine, Surgery and Pharmacy, University of Sassari, 07100 Sassari, Italy; dpasero@uniss.it; 12 Intensive Care Unit, Civil Hospital of Alghero, 07041 Alghero, Italy; 13 Infectious Diseases Unit, ARNAS Civico-Di Cristina, 90127 Palermo, Italy; 14 Department of Medicine and Health Sciences “Vincenzo Tiberio”, University of Molise, 86100 Campobasso, Italy; ripab@unimol.it

**Keywords:** antibiotics, antimicrobial resistance, decolonization, prescribing behaviours, screening, surgical antibiotic prophylaxis, surgical site infections

## Abstract

**Background:** Although surgical antibiotic prophylaxis (SAP) is considered a standard of care for preventing surgical site infections, the rising incidence of antimicrobial resistance (AMR) increases the likelihood of infections caused by multidrug-resistant organisms (MDROs), which may be associated with worse surgical outcomes. **Methods:** A multidisciplinary working group was convened by the Italian Multidisciplinary Society for the Prevention of Healthcare-Associated Infections (SIMPIOS) to define key measures for optimizing SAP in the era of AMR. Selecting the most appropriate SAP in patients colonized with MDROs is a complex decision that cannot be generalized, as it depends on both host factors and the specific surgical procedure. At present, there is limited evidence of SAP in these patients. **Results:** This position paper aims to provide practical guidance for optimizing SAP in the context of an AMR era. It is structured in three sections: (1) core principles of surgical antibiotic prophylaxis; (2) the role of screening, decolonization, and targeted prophylaxis for MDROs; and (3) barriers to changing surgeons’ prescribing behaviours. **Conclusions:** The working group developed 15 recommendation statements based on scientific evidence.

## 1. Introduction

Antibiotics play an important role across the surgical pathway, being prescribed either as surgical prophylaxis or as treatment. Surgical antibiotic prophylaxis (SAP) accounts for a substantial proportion of total antibiotic use in hospitals, and represents an estimated 15% of all prescriptions in these settings [1]. SAP is considered one of the most reliable measures to prevent surgical site infections (SSIs). It helps reduce the unavoidable bacterial contamination that occurs in the surgical area during an operation [1]. In 2013, the American Society of Health-System Pharmacists, along with the Infectious Diseases Society of America, the Surgical Infection Society and the Society for Healthcare Epidemiology of America, released guidelines on SAP [2]. Similarly, the Italian Ministry of Health published in 2011 the national guidelines that, although old, focus comprehensively on SAP principles [3].

Despite the existence of such recommendations for more than a decade, inappropriate prescribing practices remain widespread, contributing to suboptimal patients’ clinical outcomes, adverse drug effects, and the development of antimicrobial resistance (AMR) [4]. AMR poses a critical threat to global health because antibiotics are losing effectiveness in both prevention and treatment of infections. The growing prevalence of extended-spectrum beta-lactamase (ESBL)-producing and carbapenem-resistant *Enterobacterales* (CRE), vancomycin-resistant *enterococci* (VRE), and methicillin-resistant *Staphylococcus aureus* (MRSA), often acquired through community transmission, challenges the efficacy of conventional SAP regimens [5,6,7]. Addressing this challenge requires a re-examination of perioperative recommendations, evaluating individual patient risk factors for multidrug-resistant organism (MDRO) colonization. Antimicrobial stewardship programs (ASPs) and infection prevention and control programs are pivotal to this effort. They are closely linked, and targeted patient screening for MDROs is becoming an important tool to identify carriers of MDROs.

Although SAP is considered a standard of care for SSI prevention [1], the rising incidence of AMR increases the likelihood of infections caused by MDROs, which may be associated with worse surgical outcomes [8]. Selecting the most appropriate SAP in patients colonized with MDROs is a complex decision that cannot be generalized, as it depends on both host factors and the specific surgical procedure. At present, limited evidence provides guidance on SAP selection in high-risk patients. A targeted SAP approach should be considered when MDROs pose a substantial SSI risk, particularly if such infections could negatively affect outcomes. In the era of AMR, avoiding unnecessary broad-spectrum antibiotic use is essential to prevent further AMR development, too.

This position paper aims to provide practical guidance for optimizing SAP in the context of an AMR era. It is structured in three sections: (1) core principles of surgical antibiotic prophylaxis; (2) the role of screening, decolonization, and targeted SAP for MDROs; and (3) barriers to changing surgeons’ prescribing behaviours.

## 2. Materials and Methods

The Italian Multidisciplinary Society for the Prevention of Healthcare-Associated Infections (SIMPIOS) is a national scientific society that has been promoting best practices in infection prevention and control for more than 20 years.

SIMPIOS convened a multidisciplinary working group to define key measures for optimizing SAP in the era of AMR. The working group developed recommendation statements based on scientific evidence retrieved from PubMed and EMBASE. Final recommendations were completed using the Grading of Recommendations Assessment, Development and Evaluation (GRADE) framework. Evidence was classified as high, moderate, low, or very low quality, while the strength of each recommendation was rated as either weak or strong. Consensus among the guideline panel was achieved through the Delphi method. Statements reaching ≥80% agreement were approved as strong recommendations. The group collaborated via email to finalize the document and approved the present manuscript.

Although coordinated by an Italian scientific society, these recommendations were developed for global applicability and should be adapted to local epidemiology, resources, and formularies across the diverse health systems.

## 3. Core Principles for Appropriate Antibiotic Prophylaxis

In addition to prescribing targeted SAP for MDROs, optimizing general SAP practices is essential to improve antibiotic prescribing and slow the emergence of AMR. Institutional strategies should include the development and implementation of ASPs, aiming to ensure the correct choice, dose, and timing of antibiotic administration. These should always be combined with strict adherence to infection prevention and control measures.

SAP is only one of several measures to prevent SSIs, and for it to be effective, all other preventive measures must be observed throughout the pre-, intra-, and postoperative phases.

Extensive research has confirmed that SAP is effective in reducing the risk of SSIs. In a large review, Bowater and colleagues [9] examined 21 meta-analyses of randomized controlled trials (RCTs) involving 48,909 patients across 250 hospitals, demonstrating that SAP significantly reduces SSIs regardless of wound classification or surgical type, with relative risk reductions ranging from 18% to 78%.

In 2008, Whitman et al. [10] highlighted the importance of standardized SAP protocols. By retrospectively evaluating 1622 consecutive patients, they assessed whether the selected antibiotic, timing of administration, and discontinuation practices aligned with recommended guidelines. Their findings indicated that even with hospital-wide educational initiatives, ensuring adherence to evidence-based SAP practices required systems that compelled surgeons to follow proper protocols.

The World Health Organization (WHO) global guidelines for SSI prevention recommend administering SAP before surgical incision, when indicated [11]. The Centers for Disease Control and Prevention (CDC) guidelines advise administering SAP agents only when justified by published clinical practice guidelines and at the correct time to ensure appropriate antibiotic concentrations at the moment of incision [12]. Similarly, the joint guidelines for SSI prevention promoted by the Society for Healthcare Epidemiology of America (SHEA), in cooperation with the Infectious Diseases Society of America (IDSA), and the Association for Professionals in Infection Control and Epidemiology (APIC) highlight strict compliance with evidence-based practices as a fundamental requirement [13]. Finally, the Italian national guidelines [3] also suggest SAP only when justified by scientific evidence and at the right time.

SAP is recommended for surgeries with a high risk of SSIs, including many clean-contaminated and contaminated procedures. It may also be necessary for certain clean surgeries where, even though the risk of infection is low, the potential consequences of an SSI could be serious, such as in surgeries involving prosthetic implants. The antibiotic selected should target the bacteria most likely to cause SSIs after a given procedure. In clean surgical fields, SSIs are typically caused by commensal skin microbiota, primarily Gram-positive cocci such as *S. aureus* or coagulase-negative *staphylococci*. In clean-contaminated (class II) and contaminated (class III) surgeries, SSIs are usually caused by microorganisms originating from the organ’s own microbiota, including *Escherichia coli*, other *Enterobacterales* or *Clostridiales*. 

As a general rule, antibiotics with the narrowest effective spectrum of activity should be administered for SAP. Broad-spectrum agents should be avoided (except in specific justified cases) to preserve their efficacy for treating postoperative infections caused by MDROs. First- and second-generation cephalosporins are the most frequently prescribed antibiotics for SAP. Cefazolin remains the first-line choice for most surgical procedures due to its proven efficacy, favourable pharmacokinetics, appropriate spectrum, safety profile and low cost [2]. For elective colorectal surgery, cefazolin is often associated with metronidazole to ensure coverage against anaerobic bacteria. Cefoxitin, even if active against both anaerobic and aerobic Gram-negative bacteria, has largely been replaced by cefazolin plus metronidazole due to its shorter half-life. SHEA/IDSA/APIC guidelines recommend taking a detailed allergy history [14], as self-reported beta-lactam allergies are associated with a higher SSI risk due to the use of less effective alternative agents. 

Evidence does not endorse the routine use of vancomycin for SAP [2]. Its prescription is adequate for patients known to carry MRSA. However, even if vancomycin is considered appropriate for MRSA carriers, it is less effective than first-generation cephalosporins in preventing SSIs caused by methicillin-susceptible *S. aureus* [2].

The SAP dose should be equivalent to therapeutic dosing, with adjustments for special populations such as obese patients and, in some cases, transplant recipients. While the WHO does not address dosing [11], the CDC notes that there is insufficient evidence from RCTs to evaluate weight-adjusted dosing [12]. Conversely, the SHEA/IDSA/APIC guidelines, consistent with pharmacokinetic principles, recommend higher doses in obese patients (e.g., 3 g of cefazolin for those weighing more than 120 kg), supported by retrospective studies [13].

SAP should be administered at the correct time to ensure adequate tissue concentrations from the beginning of surgery [14,15]. In 2017, a meta-analysis by de Jonge et al. [14] evaluated the correct timing of SAP comparing different intervals of administration time. Fourteen studies (54,552 patients) were included. All were observational. No major differences were observed when SAP was given 120–60 min before incision compared with administration in the 60 min immediately before surgery. However, the meta-analysis showed that giving SAP after incision doubled the risk of SSIs, while administration more than 120 min beforehand increased the risk fivefold. According to WHO guidelines, SAP should be administered within 120 min before incision, taking into account the antibiotic’s half-life [11]. However, pharmacokinetic considerations suggest that most antibiotics, such as cefazoline, should be administered within 60 min of incision [2], while antibiotics with longer half-lives and longer infusion times, such as vancomycin, require initiation 2 h before the incision [2]. The SHEA/IDSA/APIC guidelines recommend starting SAP within 60 min, even if some studies suggest that administering antibiotics within 0–30 min before incision is more effective than administering them within 30–60 min [13]. In addition, these guidelines emphasize that the administration of vancomycin and fluoroquinolone should start within 120 min before incision [13].

Intraoperative re-dosing is advised for procedures lasting more than two antibiotic half-lives or associated with significant blood loss. For example, cefazolin (half-life ~2 h) should be re-dosed after ~4 h, while cefoxitin (half-life 50–60 min) should be re-dosed after ~2 h. While the CDC makes no recommendation due to insufficient RCT evidence [12], a recent meta-analysis confirms reduced SSI rates with intraoperative re-dosing [16]. The analysis included two randomized controlled trials and eight cohort studies. Despite variations in the antibiotics used, perioperative re-dosing of prophylactic antibiotics was shown to lower SSI rates compared to administering only a single preoperative dose across all types of surgery. In Table 1 we report the characteristics of the common antibiotics used for SAP.

SAP is intended solely to prevent SSIs caused by intraoperative contamination, not to treat postoperative infections. Antibiotics should be discontinued once the incision is closed. WHO, CDC, SHEA/IDSA/APIC, guidelines as well as the Italian guidelines for SAP all strongly advise against postoperative continuation [3,11,12,13], as it provides no SSI reduction, even with drains in place, but increases risks such as AMR, *Clostridioides difficile* infection, and acute kidney injury [17]. A 2020 meta-analysis of 83 RCTs confirmed no benefit of postoperative continuation when standard infection prevention measures are followed [18]. No conclusive evidence of a benefit of postoperative continuation of SAP compared to withdrawal was demonstrated. When standard infection prevention and control measures were applied, extending SAP after surgery offered no added advantage in lowering SSI rates.

Oral surgical antibiotic prophylaxis in colorectal surgery involves preoperative administration of oral antibiotics, often combined with mechanical bowel preparation (diet and cathartics), to reduce SSI risk. While common in North America, mechanical bowel preparation is less frequent in Europe due to ERAS^®^ protocols discouraging routine bowel preparation [19]. Meta-analyses [20,21,22] and RCTs [23] suggest that the association of oral antibiotic prophylaxis combined with mechanical bowel preparation can guarantee a protective effect for SSIs occurring. To minimize disruption of the colonic microbiome, oral prophylaxis should generally be limited to ≤24 h. Further high-quality trials are needed to determine optimal dosing and combinations [24].

### Recommendations

**(1) We recommend SAP in most clean-contaminated surgical procedures associated with a high incidence of SSIs. It is also indicated in some clean procedures in which SSIs, although unlikely, may have major consequences, such as in procedures with prosthetic implants** (Low quality of evidence, strong recommendation).

**(2) We recommend that the antibiotic of choice for SAP should be active against the bacteria that most commonly cause SSI after the specific procedure** (Low quality of evidence, strong recommendation).

**(3) We recommend taking a detailed allergy history for SAP antibiotics** (Low quality of evidence, strong recommendation).

**(4) We recommend the administration of antibiotic prophylaxis within 120 min before surgical incision, based on the half-life of the antibiotic, 30/60 min for common antibiotics (first- and second-generation cephalosporins) used in clinical practice** (Very low quality of evidence, strong recommendation). 

**(5) We recommend re-administering an antibiotic intraoperatively as prophylaxis for procedures that exceed the sum of two antibiotic half-lives or for procedures with significant associated blood loss** (Moderate quality of evidence, strong recommendation).

**(6) We recommend against prolonging the administration of SAP after the operation to prevent SSI** (Moderate quality of evidence, strong recommendation).

**(7) We recommend oral antibiotic prophylaxis (for no more than 24 h), associated with intravenous SAP, in elective colorectal surgery if mechanical preparation is performed** (Moderate quality of evidence, strong recommendation).

## 4. Screening, Decolonization, and Targeted SAP in Patients Colonized with Multidrug-Resistant Bacteria

The incidence of MDROs is increasing worldwide, threatening the effectiveness of SAP and raising the risk of SSIs. Understanding MDROs is essential to optimize the outcomes of SAP. However, there is a lack of consolidated evidence supporting the use of alternative broad-spectrum or targeted antibiotics in patients colonized with such pathogens [25]. Moreover, the inappropriate use of broad-spectrum antibiotics without a clear indication provides no benefit to patient care and may be harmful. In recent years, as part of infection prevention and control strategies to limit AMR transmission in the perioperative setting, attention has shifted toward screening patients before surgery and/or hospital admission.

Preoperative screening aims to detect the presence of antibiotic-resistant organisms in surgical patients. This approach can be critical to preventing SSIs caused by these pathogens, which are more challenging to treat and often associated with more severe complications.

### 4.1. Gram-Positive Bacteria

In the context of Gram-positive bacteria, preoperative screening can be highly effective as it enables targeted decolonization. Preoperative screening for *S. aureus* nasal carriage is typically performed using PCR-based rapid tests (e.g. Xpert^®^ SA Nasal Complete, Cepheid, Sunnyvale, CA, USA ) for timely results, or by culturing on selective chromogenic media (e.g. ChromID^®^ *S. aureus*, bioMérieux, Marcy-l’Étoile, France). The choice of method depends on the required turnaround time and local laboratory resources.

A large proportion of SSIs are attributable to *S. aureus*. As early as 2013, a meta-analysis of 39 studies evaluated the effectiveness of combined decolonization and prophylaxis to reduce Gram-positive SSIs after cardiac or orthopaedic surgery. The results showed that nasal decolonization significantly reduced *S. aureus*-associated SSIs, and that glycopeptides were significantly protective against MRSA-associated SSIs compared with beta-lactams. Furthermore, combining decolonization with glycopeptide SAP exclusively for MRSA-colonized patients had a strong protective effect [26].

WHO guidelines for SSI prevention [11] recommend screening and decolonization for MRSA before major orthopaedic and cardiac surgery. Specifically, topical 2% mupirocin ointment, with or without chlorhexidine soap, is recommended for nasal carriers of *S. aureus*. The review supporting this recommendation included six RCTs (n = 2385 patients) comparing mupirocin ± chlorhexidine with placebo or no treatment, showing a significant reduction in SSI rates in nasal carriers, particularly in cardiothoracic and orthopaedic surgery. Similarly, the SHEA/IDSA/APIC guidelines [13] recommend preoperative decolonization for *S. aureus* in orthopaedic and cardiothoracic procedures, and in other high-risk surgeries involving prosthetic materials.

Recent meta-analyses reinforce these findings. A 2021 meta-analysis of 12 studies in joint arthroplasty found that screening and decolonization reduced overall SSIs, methicillin-susceptible *S. aureus* (MSSA)-associated SSIs, and MRSA-associated SSIs [27]. Another meta-analysis focusing on elective arthroplasty reported increased SSI risk in patients who did not undergo screening and decolonization [28]. The latest European Society of Clinical Microbiology and Infectious Diseases/European Committee on Infection Control (ESCMID/EUCIC) guidelines recommend *S. aureus* screening before high-risk operations such as cardiothoracic and orthopaedic surgery, and the use of vancomycin SAP in MRSA carriers undergoing these procedures [29]. No recommendations were made for VRE because of insufficient evidence. 

The RCT by Bode et al., which enrolled 808 patients undergoing mainly cardiothoracic or orthopaedic surgery, demonstrated that rapid identification and decolonization with mupirocin plus chlorhexidine reduced the number of SSIs caused by *S. aureus* [30]. In the mupirocin–chlorhexidine group, the incidence of *S. aureus* infection was 3.4%, compared to 7.7% in the placebo group, corresponding to a relative risk of 0.42 (95% CI, 0.23–0.75). The use of mupirocin–chlorhexidine was particularly effective in reducing deep SSIs, demonstrating a relative risk of 0.21 (95% CI, 0.07–0.62). No statistically significant difference was observed in in-hospital mortality between the two study groups. Notably, the median time to onset of SSIs was shorter among patients receiving placebo compared with those receiving mupirocin–chlorhexidine (*p* = 0.005).

Implementation of screening programs should consider laboratory workload, local epidemiology, and coordination with antimicrobial stewardship and infection control teams. Routine vancomycin SAP is not recommended and should be reserved for MRSA carriers or patients at high risk of MRSA exposure, particularly in prosthetic surgeries [2,11].

The growing problem of mupirocin resistance and treatment failures underscores the need for alternative options in decolonization. Evidence from both preclinical and clinical research, along with official guidelines, supports the use of a 5–10% intranasal povidone-iodine solution for five days, particularly before major orthopedic surgery, as either an alternative or an adjunct to mupirocin [31,32]. This approach is especially valuable when rapid, short-term effectiveness is required, resistance to mupirocin is prevalent, or a non-antibiotic option is preferred. 

#### Recommendations

**(8) We recommend a nasal swab for screening and subsequent decolonisation of *S. aureus* for patients before any orthopaedic prosthetic and cardiac surgery procedure** (Moderate quality of evidence, strong recommendation).

**(9) We recommend targeted SAP with vancomycin in all patients colonized with MRSA (even if already decolonized), administering it 120 min before surgical incision** (Moderate quality of evidence, strong recommendation).

### 4.2. Gram-Negative Bacteria

The prevalence of patients colonized by extended-spectrum beta-lactamase (ESBL)-producing *Enterobacterales* has risen worldwide in both healthcare and community settings. Unlike *S. aureus*, gastrointestinal colonization by these organisms makes decolonization difficult, if at all possible.

WHO guidelines [11] make no recommendations on ESBL screening, due to the absence of high-quality studies, even in countries with high prevalence. The need for robust RCTs is emphasized to inform SAP strategies, prevent inappropriate broad-spectrum antibiotic use, and limit the spread of AMR. In 2023, ESCMID/EUCIC published guidelines suggesting [33]: (a) rrectal screening and targeted SAP for fluoroquinolone-resistant *Enterobacterales* before transrectal prostate biopsy; (b) rectal screening and targeted SAP for ESBL-producing *Enterobacterales* before colorectal surgery; and (c) screening for carbapenem-resistant *Enterobacterales* and *Acinetobacter baumannii* before transplantation, guided by local epidemiology.

Observational studies indicate that ESBL colonization increases SSI risk in colorectal surgery [34,35,36], with carriers having up to double the risk compared to non-carriers. Dubinsky-Pertzov et al. published a prospective cohort study in 2019 [34] including 3600 patients from three hospitals in Israel, Switzerland and Serbia who were screened for ESBL-producing *E. coli* before colorectal surgery and given cephalosporin-based SAP, showing significantly higher SSIs in carriers compared to non-carriers (24.8% vs. 11.1%, *p* < 0.001). Multivariate analysis demonstrated that being a carrier of ESBL-producing *Escherichia coli* was an independent predictor associated with a two-fold increased risk of SSIs. Another prospective cohort study, published in 2019 [35], included patients with gastrointestinal and gynecologic malignancies who were admitted to the hospital for elective surgery. Rectal swabs were obtained at admission and subsequently every five days postoperatively. The study enrolled 171 patients. Among these patients, 30 (17.5%) were found to carry ESBL-producing *Enterobacterales* at admission, increasing to 37 (21%) over the course of hospitalization. The incidence of SSI was 14.6% (25 patients). SSIs were observed in 10 of 37 colonized patients (27%) compared with 15 of 134 non-colonized patients (11%) (RR 2.163; 95% CI: 1.201–3.897; *p* = 0.016). Additionally, 5 patients developed bloodstream infections [35]. However, routine preoperative ESBL screening does not enable targeted decolonization and should be based on laboratory capacity and the risk of unnecessary broad-spectrum antibiotic use, particularly carbapenems. Risk-based screening, guided by factors such as prior hospitalization, antibiotic use, and comorbidities [37], may be more appropriate. 

Evidence on targeted SAP in ESBL carriers is limited. As a general rule, it is essential to select antibiotics with the narrowest effective spectrum. Broad-spectrum agents should generally be avoided for SAP, since they may be needed later if the patient develops a postoperative MDRO infection. An interventional study replacing cephalosporin-based SAP with ertapenem in carriers reduced ESBL-related SSI rates [38]. During the baseline period, departmental recommendations advised using a cephalosporin combined with metronidazole for SAP. In the intervention period, the protocol was updated so that patients colonized with ESBL-producing *Enterobacterales* received ertapenem instead. The overall SSI rates were 22.7% in the baseline phase and 15.8% in the intervention phase, while SSIs specifically caused by ESBL-producing *Enterobacterales* decreased from 6.5% to 0.9%. However, the methodological limitations of the study were stressed [39] in a commentary to the article. Another study, conducted within a quality improvement initiative on SAP for ESBL-producing *Enterobacterales* carriers undergoing colorectal surgery, evaluated patients 4–6 days postoperatively for colonization with ESBL-producing *Enterobacterales*, other third-generation cephalosporin-resistant *Enterobacterales*, and CREs. Among ESBL-producing *Enterobacterales* carriers, the incidence of short-term postoperative colonization with CREs and other third-generation cephalosporin-resistant *Enterobacterales* was significantly lower in those who received ertapenem prophylaxis compared with those given cephalosporin–metronidazole prophylaxis [40].

Experts generally agree that carbapenems must not be used routinely for SAP, and their routine use should be discouraged. Current consensus is to reserve ertapenem only for selected high-risk ESBL carriers or cases where SSIs have a very severe impact on outcomes. CRE remain a major global concern due to limited treatment options. 

The carbapenemase enzymes NDM (New Delhi metallo-β-lactamase) and OXA-48 are now recognized as two of the most widespread resistance mechanisms in *K. pneumoniae*. Their occurrence has increased steadily in recent years, contributing to the global rise in carbapenem-resistant infections [41]. These enzymes enable the bacteria to inactivate a broad range of antibiotics. As a result, infections caused by NDM- and OXA-48-producing *K. pneumoniae* are extremely difficult to treat, with only a few remaining therapeutic options. The rapid dissemination of these resistant strains poses a significant public health concern, underscoring the importance of adhering to strict infection control practices, implementing antimicrobial stewardship, and maintaining ongoing global surveillance. Preoperative screening is primarily valuable for infection control, with targeted SAP recommended only in selected high-risk cases, such as immunosuppressed patients or those undergoing transplantation.

Fluoroquinolone resistance has undermined traditional prophylaxis for transrectal prostate biopsy. The European Association of Urology recommends pre-screening for fluoroquinolone resistance and targeted SAP with fosfomycin where resistance rates are high [42], supported by meta-analytic evidence showing improved outcomes compared with ciprofloxacin [43].

#### Recommendations

**(10) We recommend that a rectal swab for screening faecal colonisation with ESBL-producing *Enterobacterales* before colo-rectal surgery should be well-adjusted to laboratory workloads and should be based on an individual assessment of the risk of infection and its possible impact on the patients, and the patient’s risk of being colonized** (Low quality of evidence, strong recommendation).

**(11) We recommend that carbapenems not be used routinely for SAP. We suggest reserving targeted SAP with ertapenem before colo-rectal surgery only in selected patients colonized by ESBL-producing *Enterobacterales* at high risk of SSIs or in these patients in whom SSIs can strongly impact their outcomes** (Low quality of evidence, weak recommendation).

**(12) We recommend that screening for faecal colonisation with carbapenem-resistant *Enterobacterales* and *Acinetobacter baumannii* before digestive surgery should be based on an individual assessment of the risk of infection and the patient’s risk of being colonized** (Very low quality of evidence, strong recommendation).

**(13) We recommend targeted SAP for patients colonized by carbapenem-resistant *Enterobacterales* and *Acinetobacter baumannii*, only in immunosuppressed patients and before transplantation** (Very low quality of evidence, strong recommendation).

## 5. Behavioural Changes Across the Surgical Pathway

Several studies have described the power dynamics within surgical teams that influence antibiotic decision-making, potentially limiting integrated prescribing practices along the surgical pathway. Poor awareness among surgeons regarding antibiotic prescribing has been reported [44,45]. In a qualitative study by Charani et al., based on face-to-face interviews with six surgical teams at a London teaching hospital, surgeons often described antibiotic prescribing as a “non-surgical” intervention [44].

In 2019, an ethnographic qualitative study examined differences in antibiotic decision-making between acute surgical and medical teams [46]. Surgeons typically prioritize surgical outcomes, and the fear of poor postoperative results often outweighs concerns about the adverse consequences of inappropriate antibiotic prescribing. This mindset can lead to prolonged and unnecessary antibiotic use—either by extending prophylaxis beyond recommended durations, failing to discontinue antibiotics after surgery, or continuing therapeutic courses for many days after adequate source control.

To improve quality and safety initiatives, it is crucial to consider both structural and cultural determinants influencing antibiotic use across the surgical pathway. Multiple factors, such as diagnostic uncertainty, fear of clinical failure, time pressure, and organizational constraints, can complicate prescribing decisions [47]. Cognitive dissonance, where clinicians recognize the need for action but fail to implement it, further challenges behaviour change [47].

Raising surgeons’ awareness of the importance of appropriate antibiotic use is essential. Clinical guidelines can help reduce unwarranted practices, translate evidence into practice, and enhance healthcare quality and safety. They also serve as educational and training tools for health professionals. However, guidelines are not always directly applicable in all contexts; adapting them locally—through protocols, bundles, checklists, and visual reminders—can improve acceptance and adherence.

In a collaborative environment, translating guidelines into a local protocol for SAP, with clearly defined responsibilities, should be a core component of any ASP. As surgeons are ultimately responsible for antibiotic prescriptions, their active involvement in the development and implementation of local protocols can foster a cultural shift towards adherence, which is often linked to improved outcomes.

The most effective ASPs in hospital settings are comprehensive and multidisciplinary. Promoting a safety-oriented hospital culture in which surgeons are encouraged, rather than coerced, to follow stewardship measures is essential. A strong patient safety climate is a cornerstone of healthcare quality in hospital settings.

A prospective audit and feedback strategy is one of the most widely used stewardship interventions [48]. In the surgical pathway, its main advantage is that it preserves surgeons’ prescribing autonomy, making it more acceptable and less prone to active resistance. This approach also offers educational opportunities through feedback and can be tailored to the hospital’s resources. In surgical settings, prospective audit and feedback have been associated with earlier detection of inappropriate empirical antibiotic therapy, reduced length of stay, and shorter antibiotic courses [49,50].

Key performance indicators (KPIs) are a practical way to track adherence and improvement in SAP, and can be used in the audit strategy. Based on guidelines [2,3,11,12,13] and stewardship principles, we report below the core KPIs for SAP:

- Appropriateness of indication.
  - Percentage of surgical procedures with SAP prescribed according to evidence-based guidelines (only when indicated).
- Choice of antibiotic.
  - Percentage of patients receiving the recommended first-line antibiotic for the specific procedure (e.g., cefazolin for most clean/clean-contaminated surgeries).
  - Percentage of patients unnecessarily prescribed broad-spectrum agents (should be as low as possible).
- Timing of administration.
  - Percentage of patients receiving SAP within the optimal window before incision:
    - within 30–60 min for most antibiotics (e.g., cefazolin).
    - within 120 min for agents requiring longer infusion (e.g., vancomycin).
- Adequacy of dose.
  - Percentage of patients receiving the correct dose based on guidelines.
- Intraoperative re-dosing.
  - Percentage of prolonged procedures (>2 half-lives of antibiotic or significant blood loss) with correct re-dosing.
- Duration of prophylaxis.
  - Percentage of cases where SAP is discontinued at wound closure.
  - Percentage of cases with inappropriate postoperative continuation (>24 h).
- Screening and targeted prophylaxis (when indicated)
  - Percentage of high-risk patients screened for *S. aureus* before orthopaedic/cardiac surgery.
  - Percentage of MRSA carriers receiving vancomycin for SAP.
- Compliance with local protocols
  - Overall adherence rate to hospital SAP protocols (combined indicator of indication, timing, agent, dosing, duration).
- Outcome indicators
  - SSI rate per procedure type.
  - SSI rate among patients receiving guideline-compliant SAP vs. non-compliant SAP.

Finally, identifying a local opinion leader, or “champion” can be a powerful driver of change. The concept has been successfully applied to surgical safety initiatives such as checklists. A “surgeon champion” can influence peers at a professional level, promoting best practices while maintaining close collaboration with both the infection prevention and control team and the antimicrobial stewardship team. This peer-led approach can be instrumental in achieving sustainable behavioural change. 

**Figure 1:**
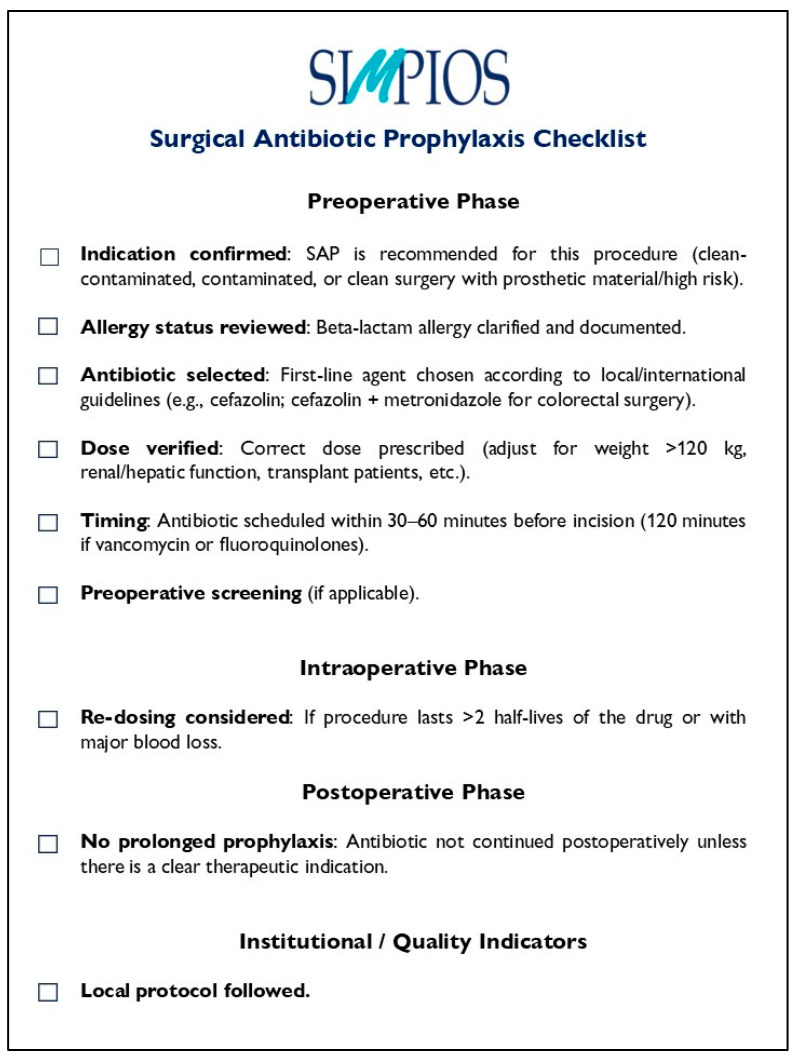
A proposal for a surgical antibiotic prophylaxis checklist.

In Figure 1, we suggest an example of SAP checklist which surgeons can use in their clinical practice.

### Recommendations

**(14) We recommend that each institution develops a local protocol for proper surgical prophylaxis, based on international guidelines** (very low quality of evidence, strong recommendation).

**(15) To change surgeons’ behaviours in prescribing practices, we recommend a multidisciplinary approach, including an audit and feedback strategy** (very low quality of evidence, strong recommendation).

## 6. Conclusions

SAP remains an essential measure to prevent surgical site infections, but its effectiveness is increasingly challenged by the global rise in AMR. Inappropriate prescribing practices not only reduce the benefits of SAP but also accelerate the spread of multidrug-resistant organisms. Optimizing SAP therefore requires a dual approach: reinforcing adherence to established principles of prophylaxis while carefully integrating targeted strategies for patients colonized with MDROs.

Evidence supports the use of narrow-spectrum antibiotics, correct timing, weight-adjusted dosing, intraoperative re-dosing when indicated, and discontinuation after wound closure. Screening and decolonization strategies, particularly for *S. aureus*, have proven effective in high-risk surgeries such as orthopaedic and cardiac procedures. For Gram-negative bacteria, including ESBL- and carbapenem-resistant *Enterobacterales*, evidence remains limited, and broad-spectrum prophylaxis should be reserved only for carefully selected high-risk patients.

Beyond clinical decision-making, behavioural and cultural factors strongly influence SAP practices. Surgeons’ perceptions of antibiotic prescribing as secondary to surgical care, combined with fear of poor outcomes, often lead to unnecessary or prolonged antibiotic use. Effective antimicrobial stewardship programs, multidisciplinary collaboration, locally adapted protocols, audit-and-feedback systems, and the engagement of “surgeon champions” are essential to promote sustained behavioural change.

In the AMR era, the goal is to ensure that SAP remains effective in preventing infections while minimizing unnecessary antibiotic exposure. This requires balancing patient safety with stewardship principles, tailoring SAP to epidemiological risk, and fostering a culture of shared responsibility across the surgical pathway.

In Table 2, we report the 15 recommendation statements based on scientific evidence expressed by the SIMPIOS multidisciplinary working group in order to provide practical guidance for optimizing SAP in the context of an AMR era.

Finally, although coordinated by an Italian scientific society, this document is intended as a globally oriented framework for SAP in the AMR era, adaptable, resource-aware, and aligned with international standards to support implementation in diverse settings.

## Figures and Tables

**Table 1:** Characteristics of the common antibiotics used for surgical antibiotic prophylaxis.

Antibiotic	Adult Dose	Pediatric Dose	Redosing Interval	Time of Administration
Cefazolin	2 g IV (3 g if >120 kg)	30 mg/kg (max 2 g)	q4h	Within 60 min before incision
Cefuroxime	1.5 g IV	50 mg/kg	q4h	Within 60 min before incision
Cefotetan	2 g IV	40 mg/kg	q6h	Within 60 min before incision
Cefoxitin	2 g IV	40 mg/kg	q2h	Within 60 min before incision
Metronidazole	500 mg IV	15 mg/kg	No redose needed	Within 60 min before incision
Clindamycin	900 mg IV	10 mg/kg	q6h	Within 60 min before incision
Vancomycin	15 mg/kg IV (≈1–1.5 g)	15 mg/kg	No redose needed (long half-life)	Begin infusion within 120 min before incision
Ertapenem	1 g IV	15 mg/kg (max 1 g)	No redose needed (long half-life)	Within 60 min before incision

IV: intravenous.

**Table 2:** Recommendation statements expressed by the SIMPIOS multidisciplinary working group.

	Recommendation Statements
**General core principles**	1. **Indication for SAP** We recommend SAP in most clean-contaminated surgical procedures as-sociated with a high incidence of SSIs. It is also indicated in some clean procedures in which SSIs, although unlikely, may have major consequences, such as in procedures with prosthetic implants *(Low-quality evidence, strong recommendation).*
	2. **Choice of antibiotic** We recommend that the antibiotic of choice for SAP should be active against the bacteria that most commonly cause SSI after the specific procedure *(Low-quality evidence, strong recommendation).*
	3. **Allergy assessment** We recommend taking a detailed allergy history for SAP antibiotics *(Low-quality evidence, strong recommendation).*
	4. **Timing of administration** We recommend the administration of antibiotic prophylaxis within 120 min before surgical incision, based on the half-life of the antibiotic, 30/60 min for common antibiotics (first- and second-generation cephalosporins) used in clinical practice *(Very low-quality evidence, strong recommendation).*
	5. **Intraoperative re-administration** We recommend re-administering an antibiotic intraoperatively as prophylaxis for procedures that exceed the sum of two antibiotic half-lives or for procedures with significant associated blood loss *(Moderate-quality evidence, strong recommendation).*
	6. **Duration** We recommend against prolonging the administration of SAP after the operation to prevent SSI *(Moderate-quality evidence, strong recommendation).*
	7. **Oral SAP in colorectal surgery** We recommend oral antibiotic prophylaxis (for no more than 24 h), associated with intravenous SAP, in elective colorectal surgery if mechanical preparation is performed *(Moderate-quality evidence, strong recommendation).*
**Screening, decolonization and** **targeted SAP**	8. **Screening for *S. aureus*** We recommend a nasal swab for screening and subsequent decolonisation of *S. aureus* for patients before any orthopaedic prosthetic and cardiac surgery procedure *(Moderate-quality evidence, strong recommendation)*.
	9. **Targeted SAP for MRSA** We recommend targeted SAP with vancomycin in all patients colonized with MRSA (even if already decolonized), administering it 120 min before surgical incision *(Moderate-quality evidence, strong recommendation).*
	10. **Screening for ESBL-producing *Enterobacterales* (before colo-rectal surgery)** We recommend that a rectal swab for screening faecal colonisation with ESBL-producing *Enterobacterales* before colo-rectal surgery should be well-adjusted to laboratory workloads and should be based on an individual assessment of the risk of infection and its possible impact on the patients, and the patient’s risk of being colonized *(Low-quality evidence, strong recommendation).*
	11. **Targeted SAP for ESBL-producing *Enterobacterales*** We recommend that carbapenems not be used routinely for SAP. We suggest reserving targeted SAP with ertapenem before colo-rectal surgery only in selected patients colonized by ESBL-producing *Enterobacterales* at high risk of SSIs or in these patients in whom SSIs can strongly impact their outcomes *(Low-quality evidence, weak recommendation).*
	12. **Screening for carbapenem-resistant *Enterobacterales* and *A. baumannii*** We recommend that screening for faecal colonisation with carbapenem-resistant *Enterobacterales* and *A. baumannii* before digestive surgery should be based on an individual assessment of the risk of infection and the patient’s risk of being colonized *(Very low-quality evidence, strong recommendation).*
	13. **Targeted SAP for carbapenem-resistant *Enterobacterales* and *A. baumannii*** We recommend targeted SAP for patients colonized by carbapenem-resistant *Enterobacterales* and *A. baumannii*, only in immunosuppressed patients and before transplantation *(Very low-quality evidence, strong recommendation).*
**Behaviours’ changes across the surgical pathway**	14. **Local protocols** We recommend that each institution develop a local protocol for proper surgical prophylaxis, based on international guidelines *(Very low-quality evidence, strong recommendation).*
	15. **Changing prescribing behaviour** We recommend that each institution develop a local protocol for proper surgical prophylaxis, based on international guidelines *(Very low-quality evidence, strong recommendation).*

ESBL: extended-spectrum beta-lactamase; MRSA: methicillin-resistant *Staphylococcus aureus*; SAP: surgical antibiotic prophylaxis; SSI: surgical site infection.

## Data Availability

Not applicable.

## Abbreviations

AMR	Antimicrobial resistance
APIC	Association for Professionals in Infection Control and Epidemiology
ASP	Antimicrobial stewardship program
CDC	Centers for Disease Control and Prevention
CRE	Carbapenem-resistant *Enterobacterales*
ESBL	Extended-spectrum beta-lactamase
ESMID	European Society of Clinical Microbiology and Infectious Diseases
EUCIC	European Committee on Infection Control
GRADE	Grading of Recommendations Assessment, Development and Evaluation
IDSA	Infectious Diseases Society of America
KPI	Key performance indicator
MDRO	Multidrug-resistant organism
MRSA	Methicillin-resistant *Staphylococcus aureus*
MSSA	Methicillin-susceptible *Staphylococcus aureus*
RCT	Randomized controlled trial
SAP	Surgical antibiotic prophylaxis
SIMPIOS	Italian Multidisciplinary Society for the Prevention of Healthcare-Associated Infections
SHEA	Society for Healthcare Epidemiology of America
SSI	Surgical site infection
VRE	Vancomycin-resistant *enterococci*
WHO	World Health Organization
